# *KRAS* mutation detection and prognostic potential in sporadic colorectal cancer using high-resolution melting analysis

**DOI:** 10.1038/sj.bjc.6605959

**Published:** 2010-10-19

**Authors:** V Deschoolmeester, C Boeckx, M Baay, J Weyler, W Wuyts, E Van Marck, M Peeters, F Lardon, J B Vermorken

**Affiliations:** 1 Laboratory of Cancer Research and Clinical Oncology, Department of Medical Oncology, University of Antwerp/Antwerp University Hospital, Wilrijk 2610, Belgium; 2 Department of Epidemiology and Social Medicine, University of Antwerp, Wilrijk 2610, Belgium; 3 Department of Medical Genetics, University of Antwerp/Antwerp University Hospital, Wilrijk 2610, Belgium; 4 Department of Pathology, University Hospital of Antwerp, Edegem 2650, Belgium

**Keywords:** colorectal cancer, high-resolution melting analysis, prognosis, KRAS, survival analysis

## Abstract

**Background:**

The development of targeted therapies has created a pressing clinical need for molecular characterisation of cancers. In this retrospective study, high-resolution melting analysis (HRMA) was validated and implemented for screening of 164 colorectal cancer (CRC) patients to detect *KRAS* hot-spot mutations and to evaluate its prognostic value. Direct sequencing was used to confirm and characterise HRMA results.

**Methods:**

After establishing its sensitivity, HRMA was validated on seven cell lines and inter- and intra-variation were analysed. The prognostic value of *KRAS* mutations in CRC was evaluated using survival analysis.

**Results:**

HRMA revealed abnormal melting patterns in 34.1% CRC samples. Kaplan–Meier survival curves revealed a significantly shorter overall (OS) and disease-free survival (DFS) for CRC patients harbouring a *KRAS* mutation. In the Cox regression analysis, only when colon and rectal cancer were analysed separately, *KRAS* mutation was a negative predictor for OS in patients with rectal cancer and DFS in those with stage II colon cancer.

**Conclusions:**

HRMA was found to be a valid screening method for *KRAS* mutation detection. The *KRAS* mutation came forward as a negative predictive factor for OS in patients with rectal cancer and for DFS in stage II colon cancer patients.

Among the most daunting challenges facing oncology today is that of patient selection, particularly for therapy with molecularly targeted agents (Jimeno *et al*, 2009). Hence, robust prognostic markers and markers predictive for treatment response, resistance and toxicity are necessary.

For example, the epidermal growth factor receptor (EGFR) has become an important target for treatment of metastatic colorectal cancer (mCRC), specifically with the monoclonal antibodies (mAbs) cetuximab and panitumumab (Heinemann *et al*, 2009). Several reports indicate that an increased gene copy number of *EGFR* or mutations of genes responsible for downstream signalling, especially *KRAS*, are important determinants of response or resistance to anti-EGR antibodies (Heinemann *et al*, 2009).

*KRAS* is part of a group of three homologous oncogenes and encodes a small 21 kDa protein (p21Ras) involved in the transduction of external stimuli to effector molecules across plasma membranes, downstream from the EGFR. This protein has intrinsic guanosine triphosphatase (GTPase) activity, allowing inactivation after signal transduction in the normal cellular environment. Somatic point mutations of *KRAS* occurring early in CRC tumourigenesis are thought to abolish GTPase activity, leading to a constitutive activation of *KRAS*, and inevitably to increased and unregulated cellular proliferation and malignant transformation (Adjei, 2001; Conlin *et al*, 2005). Oncogenic mutations of the *KRAS* gene are observed in ∼40% of sporadic CRC, and up to 90% of these mutations are detected in codons 12 and 13 and less frequently also in codons 61 and 63 (Heinemann *et al*, 2009). The functions of *KRAS* support its putative predictive and prognostic role in CRC, and several studies have been performed trying to illustrate this (Graziano and Cascinu, 2003).

With respect to its predictive role, several retrospective analyses of tumour samples in CRC patients receiving anti-EGFR antibody treatment have shown that patients with mutated *KRAS* did not benefit from anti-EGFR therapy (Lievre *et al*, 2006; Amado *et al*, 2008). Based on systematic reviews of the relevant literature, the American Society of Clinical Oncology suggested, in a Provisional Clinical Opinion in 2009, that when *KRAS* mutations in codon 12 or 13 were detected in patients with mCRC, such patients should not receive anti-EGFR antibody therapy as part of their treatment (Allegra *et al*, 2009). The European Medicines Agency has also recognised these findings, and indeed also restricts the use of anti-EGFR antibody therapy only to CRC patients with wild-type (wt) *KRAS* tumours (Javle and Hsueh, 2009).

With respect to its prognostic role in CRC, literature data on the impact of *KRAS* mutations on outcome has been controversial, including in those with node-negative disease, for whom a discriminator would be most useful (Jimeno *et al*, 2009, and as reviewed by Anwar *et al*, 2004; Klump *et al*, 2004; Locker *et al*, 2006).

In addition, with the advent of personalised medicine, there is a compelling need for rapid and accurate methods for detection of nucleic acid sequencing changes, such as, *KRAS* mutations, in clinical specimen (Krypuy *et al*, 2006). A wide range of mutation detection techniques exists, of which sequencing has been the gold standard (Krypuy *et al*, 2006). However, its limited sensitivity, high costs and long turnaround time have prompted the development of alternative methods for routine clinical testing that have greater diagnostic practicality for somatic mutation detection (Do *et al*, 2008). High-resolution melting analysis (HRMA) is a recently developed methodology that has enormous potential for the detection of DNA sequence changes (Do *et al*, 2008). Mutation scanning with HRMA is based on the dissociation behaviour of DNA when exposed to an increasing temperature, in the presence of intercalating fluorescent dyes. The HRMA melting profile gives a sequence-related pattern, allowing discrimination between wt sequences and homozygote–heterozygote variants (Giuliani *et al*, 2008). Owing to its high sensitivity, HRMA seems to present a more sensitive approach, allowing rapid, accurate and reliable detection of a minimal fraction of mutated cells in tumoral tissue (Giuliani *et al*, 2008; Kramer *et al*, 2009).

The aim of this study was to validate and implement HRMA to detect *KRAS* mutations in formalin-fixed paraffin-embedded (FFPE) CRC samples. In addition, the prognostic value of *KRAS* mutation was evaluated in a population of CRC patients.

## Materials and methods

### Samples and DNA extraction

Tissue samples were obtained from 164 sporadic CRC patients treated at the Antwerp University Hospital in Edegem and the St Augustinus Hospital in Wilrijk. DNA was extracted from FFPE tissue blocks as described previously (Deschoolmeester *et al*, 2006). DNA concentration and purity was defined using the Nanodrop 1000 (Isogen, Sint-Pieters-Leeuw, Belgium). Microsatellite instability (MSI) status was defined previously (Deschoolmeester *et al*, 2008).

### Assay design and PCR conditions

Primers were designed to span codons 12 and 13 of the *KRAS* gene. Primers for the 114-bp amplicon of exon 2 were 5′-GCCTGCTGAAAATGACTGAA-3′ (forward) and 5′-TTGGATCATATTCGTCCACAA-3′ (reverse). The reaction mixture was made up using 2.5 × LightScanner Mastermix (Idoha Technology Inc., Salt Lake City, UT, USA), 1.65 mM MgCl_2_, 5 *μ*M of each sense and antisense primer, 4% (v/v) DMSO, 2 *μ*l genomic DNA or 20 ng cell line DNA and water in a total volume of 10 *μ*l.

The PCR cycling was performed on the Rapid Cycler Instrument 2 (Idoha Technology Inc.), whereas HRMA was performed on the HR/1 High-Resolution Melter (Idoha Technology Inc.) and measured by the HR/1 Instrument Control software. The 114-bp amplicon was run according to the following conditions: one cycle of 95°C for 30 s and 45 cycles in the following sequence: 95°C for 10 s, 65°C for 10 s and 74°C for 2 s. The results were analysed using the HR/1 Melt Analysis Tool software (Idoha Technology Inc.).

### HRMA sensitivity testing

High-resolution melting analysis sensitivity testing was conducted by mixing a series of dilutions of 50, 25, 12.5, 6 and 3% of mutant *KRAS* DNA from A549 (G12S, homozygous mutation in codon 12) within wt *KRAS* DNA from CAL27. In addition, these cell lines were also used as positive and negative controls.

Subsequently, the HRMA of *KRAS* mutations was validated in a set of DNA obtained from several cell lines (Table 1) with or without a known *KRAS* mutation.

### DNA sequencing

After HRMA, the PCR products with a deviating pattern were separated on a 2% low melting point agarose gel (Ultra Pure, Gibco BRL, Merelbeke, Belgium) during 60 min on 50 V. After separation, the desired bands were excised from the gel and the DNA was isolated and purified using spin procedure for agarose gels (GenElute Gel Extraction Kit, Sigma, Bornem, Belgium). The purified PCR product was then used as template in cycle sequencing using the Big Dye Terminator v1.1 kit (Applied Biosystems, Foster City, CA, USA). The reaction mixture consisted of 1.1 × sequencing buffer, 0.2 *μ*l Big Dye mix, 625 nM primer and 1 *μ*l of cleaned template in a total volume of 4 *μ*l. The forward and reverse reactions were run on a Rapid Cycler Instrument 2 (Idoha Technology Inc.) according to the following protocol: one cycle of 95°C for 30 s and 25 cycles in the following sequence: 96°C for 10 s, 50°C for 5 s and 60°C for 2 min. The sequencing reactions were run on a 3130 XL Genetic Analyzer (Applied Biosystems). Sequencing data was analysed using SeqScanner software v1.0 (Applied Biosystems).

### Statistical analysis

Prognostic relevance of *KRAS* mutation was assessed by survival analysis. The index date for survival time calculation was defined as the date of diagnostic confirmation for CRC. The months of observation (overall survival (OS) time) were calculated from the index date to the date of last information/death. For disease-free survival (DFS) time, the months of observation were calculated from the index date to the first date of progression or the date of last information. Survival curves were determined by using the Kaplan–Meier method and were analysed by using the log-rank test.

Possible associations between *KRAS* mutation and clinicopathological parameters of CRCs were investigated using the *χ*^2^-test or Fisher's exact test (when appropriate) for categorical variables and using Student *t*-test or Mann–Whitney *U*-test (when appropriate) for continuous variables. To assess the independent prognostic contribution of *KRAS* mutation, a multiple Cox regression analysis was conducted. In addition, a stepwise backward binary logistic regression was performed to identify which of the clinicopathological parameters had the strongest impact on survival in CRC. All analyses were conducted using SPSS (version 16.0, SPSS Inc., Brussels, Belgium). Significance for all statistics was recorded if *P*<0.05 (two tailed).

## Results

### Patient characteristics

Of the 164 CRC patients from whom tumour tissue could be obtained, most (but not all) clinical data were retrieved. Most tumours were located in the distal part of the large bowel (68.3%), and most patients had a stage II or III disease. Further details on these patients are summarised in Table 2.

### Assay sensitivity testing

**Figure 1:**
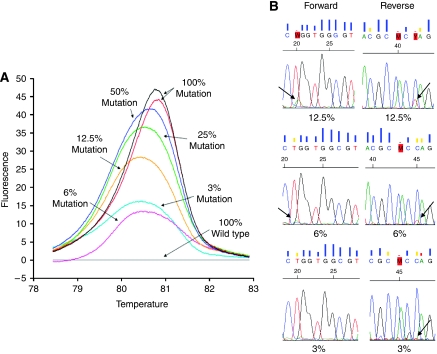
(**A**) Difference plot generated with serial dilutions of DNA from a mutated cell line in wild-type DNA to assess HRMA sensitivity. (**B**) Electropherograms of sequencing analysis for the same serial dilutions. Only 12.5% could be incontestably confirmed by sequencing analysis.

Sensitivity of the melting profile in discriminating different percentages of mutated alleles was initially evaluated by using serial dilutions of mutated DNA, derived from a cultured cell line with a known *KRAS* mutation, variably mixed with wt DNA obtained from a wt cell line. A549 DNA (G12S, homozygous mutation in codon 12) was mixed with wt CAL27 DNA in proportions of 50, 25, 12.5, 6 and 3%. The difference plot (Figure 1A) shows that HRMA was able to identify the presence of an abnormal profile in all dilutions, allowing the clear identification of mutated alleles. In addition, dideoxy sequencing analysis was performed on the 12.5, 6 and 3% dilutions using the same PCR product after melting analysis (Figure 1B). The results of HRMA were only confirmed for the 12.5% dilution by sequencing analysis in both the forward and the reverse primer set. In the case of 6% (for forward primer) and 3% (both forward and reverse primer) mutant DNA in wt DNA, sequencing analysis was not able to incontestably confirm the presence of mutant alleles, as seen by the nucleotide sequence generated by the sequencing software.

### Assay validation

**Figure 2:**
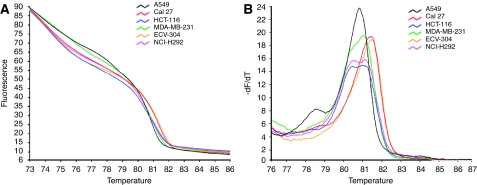
High-resolution melting analysis for different cell lines with or without a known *KRAS* mutation. (**A**) Melting curves, not normalised. (**B**) Derivative plot of the non-normalised melting curves.

**Figure 3:**
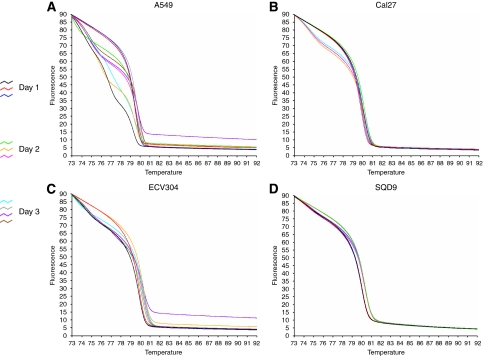
Inter- and intra-variance analysis of four different cell lines (**A**: A549, **B**: CAL27, **C**: ECV304 and **D**: SQD9) on 3 different days.

**Figure 4:**
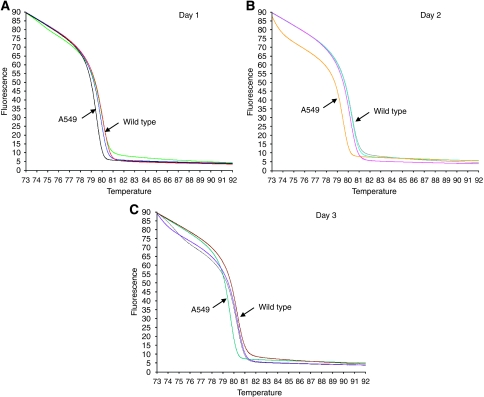
Inter- and intra-variation analysis in four different cell lines (three wild type: ECV304, CAL27 and SQD9; one mutant: A549) on 3 consecutive days (**A**: day 1, **B**: day 2 and **C**: day 3). Within each replicate of the same experiment, the mutant cell line (A549) is clearly discriminated from the three wild-type cell lines.

Cancer cell lines with or without a known *KRAS* mutation (Table 1) were first used to test the HRMA methodology. The HRMA was able to discriminate between the wt DNA and the different mutations present in the mutant cell line DNA. As seen in the melting (Figure 2A) and derivative plot (Figure 2B), HCT116, MDA-MB-231 and NCI-H292 showed typical heteroduplex melting patterns and were readily distinguishable from the wt cell lines CAL27 and ECV304. The lung cancer cell line A549 has a homozygous mutation (Table 1) and, as expected, it showed a similar shape to the wt pattern but with earlier melting of the amplified product, which is consistent with the lower thermal stability of AT base pairs relative to GC base pairs. Furthermore, dideoxy sequencing analysis confirmed the HRMA results in all cases (data not shown). In addition, an inter- and intra-variation analysis was performed on three different days in four different cell lines (A549, CAL27, ECV304 and SQD9). The results showed that inter- and intra-variation was present and could hamper the interpretation of the results (Figure 3). However, within one experiment, it was still possible to discriminate between DNA of the mutant cell line and that of the three wt cell lines (Figure 4). These results indicate that it is necessary to include positive (mutant) and negative (wt) controls in each experiment. In addition, owing to the inter-variation, it is inadvisable to compare plots generated on different days.

### *KRAS* mutation detection in CRC samples

**Figure 5:**
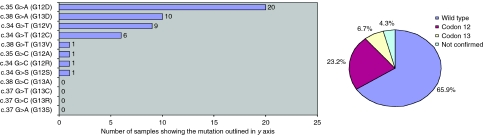
Frequencies of *KRAS* mutations based on 164 CRC samples, classified per specific mutation in codon 12 or 13.

The 114-bp amplicon was used to screen for *KRAS* mutation in codons 12 and 13 of 164 sporadic CRC samples. Aberrant curves were detected in 56 of 164 (34.1%) samples assayed. A total of 49 samples were confirmed by sequencing analysis and additionally revealed the actual mutation. Seven samples could not be confirmed by sequencing analysis, either due to lack of material or due to inconclusive results. As not all HRMA-positive samples could be confirmed by sequencing, the percentage of established *KRAS* mutations in CRC is reduced to 29.9%. As shown in Figure 5, 23.2% of the samples showed mutations in codon 12, whereas only in 6.7% of the samples, the mutations was found in codon 13. Among the different mutations, G12D substitution was the most prevalent (40.8%), followed by G13D, G12V and G12C. The other mutations were less frequently detected (Figure 5).

### Prognostic relevance of *KRAS* mutation in CRC

Follow-up for OS and DFS was available for 153 and 139 CRC patients, respectively. At the end of the observation period, 58 patients (37.9%) were deceased and 37 patients (27.0%) experienced a recurrence of the tumour. All deaths were cancer related. The median follow-up for OS and DFS was 4.7 and 4.5 years, respectively.

**Figure 6:**
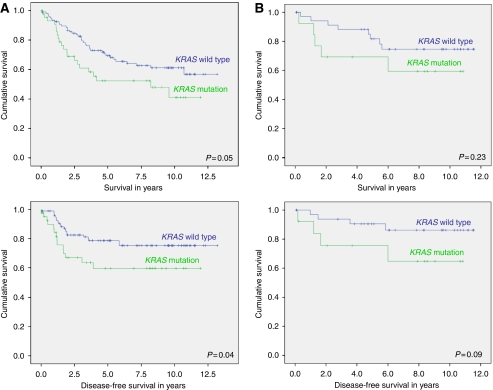
Kaplan–Meier survival analysis for *KRAS* mutation in CRC of the overall population (**A**) and of stage II patients in particular (**B**) for both overall and disease-free survival. Significance was calculated using log-rank statistic.

In the overall study population, the presence of a *KRAS* mutation was significantly associated with proximal location of the tumours (*P*=0.05). Age, gender, stage, MSI status and grade of differentiation did not seem to be correlated to the occurrence of a *KRAS* mutation. Patients with a *KRAS* mutation showed a shorter OS (HR, 1.70; *P*=0.05) and DFS (HR, 2.03; *P*=0.04) compared with patients with wt *KRAS* in the Kaplan–Meier analysis (Figure 6). The data were even more significant for those with a G12C mutation (*P*=0.04 and *P*=0.006 for OS and DFS, respectively), but the number of patients with a G12C mutation was extremely small (*n*=6). Both patients with an MSI tumour (*P*=0.05) and those with an early-stage tumour had a significantly longer OS, whereas early-stage tumours also had a significantly longer DFS (*P*<0.001). When entered into a multiple Cox regression analysis adjusting for possible important confounding factors, early stage was still significantly correlated with a longer OS (HR, 1.99; *P*<0.001) and DFS (HR, 2.35; *P*<0.001), whereas age only had a significant impact on OS (HR, 1.04; *P*<0.001). The effect of *KRAS* mutation on survival could not be confirmed in the multiple Cox regression analysis for the overall population. However, *KRAS* mutation was still a negative predictor of survival when analysed separately for rectal cancer patients (Table 3).

As mentioned earlier, the value of *KRAS* mutations to define who should benefit from adjuvant chemotherapy and who should not is especially important for stage II CRC. Therefore, a stage-dependent survival analysis was performed, which indicated that stage II patients with a *KRAS* mutation had a trend towards a worse DFS (HR, 3.06; *P*=0.09), whereas there was no significant association with a worse OS (HR, 1.97; *P*=0.23).

Interestingly, when stage II colon and rectal tumours were analysed separately, only the presence of a *KRAS* mutation in colon cancers was associated with a trend towards a worse DFS (HR, 3.64; *P*=0.07), and these results were maintained in the Cox regression analysis (HR, 4.163; *P*=0.07).

## Discussion

In the era of targeted therapy for cancer, molecular diagnosis of particular genetic markers in tumours enables a more individualised treatment of patients, as was recently shown for *KRAS* mutation status and response rate to anti-EGFR therapy in CRC (Pichler *et al*, 2009).

Although it has now been well established that *KRAS* mutation is a negative predictor for response to anti-EGFR therapy (Allegra *et al*, 2009; Balko and Black, 2009; Baynes and Gansert, 2009; Javle and Hsueh, 2009; Jimeno *et al*, 2009; Kohne and Lenz, 2009; Loupakis *et al*, 2009; Peeters *et al*, 2009; Saif and Shah, 2009), the prognostic role of *KRAS* mutations in CRC is still unclear (as reviewed by Anwar *et al*, 2004; Klump *et al*, 2004; Locker *et al*, 2006).

Given the potential impact of *KRAS* mutation detection for CRC prediction and prognosis, a reliable diagnostic test may affect future therapeutic decision making. In this study, it was demonstrated that HRMA is a reliable and sensitive technology for *KRAS* mutation detection. First, a cell-line-based model system was used to establish and optimise the *KRAS* mutation HRMA. A relative short amplicon (114-bp) was used, as it has been stated that amplicon length could influence the sensitivity of genotyping (Krypuy *et al*, 2006; Pichler *et al*, 2009). Shorter amplicon lengths have given better resolution of genotypes and increased the sensitivity of mutation detection, even down to 1% mutated cell DNA (Pichler *et al*, 2009). Our experiments on reconstituted samples, obtained by serial dilutions of a mutated cancer cell line and normal DNA, indicated the possibility of identifying at least 3% of mutated alleles in a background of wt DNA, which was the smallest dilution tested. This theoretical sensitivity seems well suited for detecting even a limited percentage of mutated alleles in a heterogeneous sample, as obtained from FFPE CRC tissues (Giuliani *et al*, 2008). These results are in line with several other studies which found that HRMA could detect 5% mutated cell DNA in a background of wt DNA (Krypuy *et al*, 2006; Do *et al*, 2008; Simi *et al*, 2008; Pichler *et al*, 2009). In comparison to sequencing analysis, HRMA seems to be more sensitive in this study, as sequencing analysis had a detection limit of 12.5% mutated alleles in a background of wt DNA. This is in agreement with the literature, as sequencing requires mutant copies to have a concentration that is at least 20–50% of any accompanying wt sequences (Ogino *et al*, 2005; Jimeno *et al*, 2009; Pichler *et al*, 2009).

Next, HRMA was validated in a set of DNA obtained from several cell lines (Table 1) with or without a known *KRAS* mutation. As reported by others (Krypuy *et al*, 2006; Simi *et al*, 2008), the heterozygous and homozygous mutations in HCT116, MDA-MB-231 and A549 could be detected with HRMA. In addition, a heterozygous *KRAS* mutation (G12S) in NCI-H292 was detected by HRMA. These results were confirmed by sequencing analysis. It might be possible that the NCI-H292 cell line acquired a *KRAS* mutation during prolonged cell culture. Therefore, the *KRAS* status needs to be examined in an independent NCI-H292 cell line to definitively confirm the presence of the heterozygous *KRAS* mutation. As mentioned in several studies (Krypuy *et al*, 2006; Do *et al*, 2008; Pichler *et al*, 2009), the presence of unspecific PCR products, primer dimers or differing salt or inhibitor concentrations may increase the spread of wt curves. Consequently, it is critical to melt highly specific PCR products. Subsequently, this study showed that inter- and intra-variation was present and could hamper the interpretation of the results on HRMA. However, within one experiment, it was still possible to clearly discriminate mutated samples from wt samples.

The *KRAS* mutations were detected and confirmed by sequencing in 49 of 164 (29.9%) CRC samples. This is in agreement with the literature in which *KRAS* mutation frequencies range from 30 to 50% (Jimeno *et al*, 2009; Peeters *et al*, 2009). The G/A transitions and G/T transversions were identified as the most frequently found type of *KRAS* mutation, as described by various studies (Samowitz *et al*, 2000; Bazan *et al*, 2005; Poehlmann *et al*, 2007; Neumann *et al*, 2009). Codon 12 harboured 23.2% of the point mutations detected, with the G12D mutation, in which glycine is replaced by aspartic acid, the most prevalent type (40.8%). These results are confirmed by Simi *et al* (2008). Although all positive sequencing results were detected by HRMA, some HRMA-positive samples could not be confirmed by sequencing. This might be explained by the fact that adverse effects of formalin fixation on DNA or Taq polymerase errors can cause PCR artefacts during amplification (Srinivasan *et al*, 2002; Do *et al*, 2008; Pichler *et al*, 2009). Unfortunately, fresh frozen tissue was not available for comparison to confirm this hypothesis. Another possibility is that some samples contained levels of mutation below the sensitivity of sequencing detection as a result of low percentage of tumour in the sample or genetic heterogeneity within the tumour (Do *et al*, 2008). However, in our HRMA, no correlation was found between low DNA concentrations and/or purity and unconfirmed positive samples. In addition, all positive samples were repeated by independent amplification to avoid false-positive results due to errors introduced by Taq polymerase.

It has been known that *KRAS* point mutations are extremely infrequent in sporadic MSI-H tumours (Ionov *et al*, 1993; Salahshor *et al*, 1999; Samowitz *et al*, 2001; Zhao *et al*, 2008). However, in several recent reports, it has been shown that the occurrence of mismatch repair (*MMR*) gene mutations at an early stage might be significant in tumourigenesis through *KRAS* mutation in MSI-H CRC. It still remains enigmatic why only the mismatch mutL homologue 1 (*MLH1*) mutation correlates with *KRAS* mutation, but *MLH1* promoter methylation does not, in spite of equally defective MMR leading to a mutator phenotype (Jass, 2002; Zhao *et al*, 2008; Kumar *et al*, 2009). In this study, *KRAS* mutation was found in only one MSI-H sample, which might be explained by the fact that our samples are derived from sporadic CRC in which *MLH1* promoter methylation is believed to be the main route of tumourigenesis. The *KRAS* mutation was associated with proximal location of the tumour. These results are confirmed by others (Elnatan *et al*, 1996; Samowitz *et al*, 2000; Andreyev *et al*, 2001; Oliveira *et al*, 2007; Ogino *et al*, 2009b). Kaplan–Meier survival analysis of the entire study population revealed a significantly shorter OS and DFS for CRC patients harbouring a *KRAS* mutation. In Cox regression, significance of *KRAS* mutation as a predictor of survival was lost. When colon tumours and rectal tumours were analysed separately, the presence of a *KRAS* mutation was associated with a worse DFS for colon cancers and a worse OS for rectal tumours in univariate analysis. In Cox regression, only the results for rectal cancer were maintained.

Stage-dependent survival analysis was performed, in particular for stage II, as the value of *KRAS* mutations to define who should receive adjuvant chemotherapy and who should not is especially important for these patients. In addition, Roth *et al* (2009) suggested that molecular markers in colon cancer have a stage-specific prognostic value and that different stages might represent different diseases rather than sequential steps in the evolution of a single disease. Kaplan–Meier survival curves revealed a trend towards a worse DFS for stage II patients harbouring a *KRAS* mutation. When analysed separately for stage II colon cancer and stage II rectal cancer, this trend was only seen in colon cancer patients, and the negative impact was maintained in the Cox regression analysis.

Previous reports have shown conflicting results concerning the relation with prognosis (Laurent-Puig *et al*, 1992; Andreyev *et al*, 2001; Losi *et al*, 2004; Roth *et al*, 2010). These contradictions are partly related to the heterogeneous nature of the relevant studies, but may also be due to the role that stage may have on the effect of genetic factors on prognosis (Cerottini *et al*, 1998). In addition, recent studies suggest that different *KRAS* gene mutations have different impacts on outcome (Cerottini *et al*, 1998; Andreyev *et al*, 2001). In this study, only G12C substitutions were significantly associated with a worse OS and/or DFS in the overall population, although caution is mandatory because some mutations were only found once. These results are in agreement with Moerkerk *et al* (1994) who identified G/T and G/C transversions in codon 12 to be associated with advanced disease. Finkelstein *et al* (1993) established a correlation between G12D mutation and haematogenous metastasis at the time of diagnosis. In the same study, G12V and G13D were found to have no impact on survival. In contrast, prognostic significance for *KRAS* codon 13 mutations has been reported in CRC (Pajkos *et al*, 2000; Samowitz *et al*, 2000; Bazan *et al*, 2002). The RASCAL II study demonstrated a significant influence on survival of only one mutation, G12V, especially in Dukes C patients (Andreyev *et al*, 1998, 2001). This was not confirmed in our study, although more G12V than G12C mutations were found. Further analyses are clearly necessary.

In contrast, recent analyses from the CALGB89803 (stage III) and PETACC-3 study (stage II and III) trials did not demonstrate *KRAS* mutation to be a prognostic marker for colon cancer patients treated with adjuvant 5FU-based chemotherapy (Tejpar *et al*, 2010). These results are in contrast to those of the RASCAL studies and also to the trend towards a worse DFS in stage II colon cancer patients found in this study. Roth *et al* (2010) and Ogino *et al* (2009a) argue that the meta-analyses of Andreyev *et al* (1998, 2001) substantially suffered from publication bias and possibly resulted in false-positive results because of the number of subset analyses. However, the patients enroled in the randomised trials (GALGB89803 and PETACC-3) may differ from the population at large, as they are selected on the basis of eligibility criteria. Recently, the MRC COIN trial could not find an improvement in the OS or progression-free survival of mCRC patients treated with cetuximab, but they did show that *NRAS*, *KRAS* and *BRAF* were strongly prognostic regardless of cetuximab treatment (Maughan *et al*, 2010).

The differences between studies might also be related to the retrospective nature of the analyses of single-arm investigations performed in small and often heterogeneous cohorts of patients in which rectal and colon tumours have been examined together. In addition, patients may not have been stratified by stage, gender or age. Thus, many have been statistically underpowered to provide meaningful results (Tejpar *et al*, 2010). However, in this study, although retrospective in nature, colon and rectal tumours were analysed separately and patients were stratified according to stage. In addition, a lack of standardisation of methodologies for marker assessment has resulted in data that are not comparable, and not all mutations within a given gene are always screened for, possibly leading to underestimation of the role of *KRAS* mutations (Tejpar *et al*, 2010). Larger prospective studies are required to provide a decisive answer, if possible.

The predictive value of *KRAS*, that is, whether or not patients will respond to anti-EGFR therapy, could not be analysed in this retrospective study, as anti-EGFR mAbs were not available for treatment of these patients at the time. In addition, only 13.7% of the study population had distant metastases, the setting for which treatment with anti-EGFR therapy has been approved (Allegra *et al*, 2009; Heinemann *et al*, 2009; Jimeno *et al*, 2009). However, of the mCRC patients in our study population, 50% had a KRAS mutation, which would render anti-EGFR therapy ineffective. This underlines the importance of *KRAS* mutation analysis.

In conclusion, HRMA was found to be a fast, efficient and reproducible screening method for *KRAS* mutation detection, using which also DNA from FFPE tissues can be tested. However, further validation studies are needed before this technique can be used in the clinical setting. The *KRAS* mutation in our retrospective study came forward as a negative predictive factor for OS in patients with rectal cancer and for DFS in stage II colon cancer patients (trend). Our data support the idea that evidence is accumulating that poor outcome could be linked to specific mutations and that specific gene mutations might have an impact on patient selection for adjuvant treatment.

## Figures and Tables

**Table 1:** Characteristics of the human cell lines used for sensitivity testing and validation of the HRM analysis technique for *KRAS* mutation detection

**Cell line**	**Origin (human)**	**Mutation**	**Reference**
A549	Lung carcinoma	*KRAS* exon 2: G12S homozygous	Krypuy *et al*, 2006
CAL27	Head and neck carcinoma		
ECV304	Bladder carcinoma		
SQD9	Head and neck squamous carcinoma		
NCI-H292	Lung carcinoma		
HCT116	Colon carcinoma	*KRAS* exon 2: G13D heterozygous	Krypuy *et al*, 2006; Simi *et al*, 2008
MDA-MB231	Breast carcinoma	*KRAS* exon 2: G13D heterozygous	Cosmic data set, Welcome Trust Sanger Institute, 2009: http://www.sanger.ac.uk/perl/genetics/CPG/core_line_viewer?action=sample&id=909960

Abbreviation: HRM=high-resolution melting.

**Table 2:** Clinical characteristics of patients analysed for the overall population and for patients with colon cancer and rectal cancer separately

	**Colon**	**Rectum**	**Overall population**
*Patient characteristics*			
Total no. of patients	103	50	164
Median age (years)	66±13	63±12	65±13
			
*Sex*			
Male	51 (49.5%)	24 (48.0%)	80 (48.2%)
Female	51 (49.5%)	26 (52.0%)	78 (48.8%)
			
*Location*			
Proximal	—	—	45 (27.4%)
Distal	—	—	112 (68.3%)
			
*Grade of differentiation*			
Poor	10 (9.7%)	3 (6.0%)	14 (8.5%)
Moderate	34 (33.0%)	18 (36.0%)	55 (33.5%)
Well	56 (54.4%)	28 (56.0%)	87 (53.0%)
			
*Stage*			
I	11 (10.7%)	8 (16%)	20 (12.2%)
II	43 (41.7%)	20 (40.0%)	69 (42.1%)
III	31 (30.1%)	14 (28.0%)	45 (27.4%)
IV	14 (13.6%)	8 (16.0%)	22 (13.4%)
			
*Therapy*			
*Neo-adjuvant*			
Yes	1 (1.0%)	19 (38.0%)	22 (13.4%)
No	89 (86.4%)	24 (48.0%)	120 (73.2%)
*Adjuvant*			
Yes	35 (34.0%)	16 (32.0%)	57 (34.8%)
No	63 (60.2%)	27 (54.0%)	92 (56.1%)
			
*MSI status*			
MSI	13 (12.6%)	0 (0.0%)	14 (8.5%)
MSS	90 (87.4%)	50 (100%)	150 (91.5%)

Abbreviations: MSI=microsatellite instability; MSS=microsatellite stability.

In 11 patients the actual location of the tumour was not specified; not all clinical characteristics were available for each patient.

**Table 3:** Survival analysis (univariate and Cox regression) for the presence of a *KRAS* mutation in the overall population and for colon cancer and rectal cancer separately

	**Colon**				**Rectum**				**Overall population**			
	***n***	**HR**	**95% CI**	***P*-value**	***n***	**HR**	**95% CI**	***P*-value**	***n***	**HR**	**95% CI**	***P*-value**
*Univariate analysis*												
OS	100	1.26	0.65–2.45	0.48	47	4.20	1.56–11.27	0.004	153	1.70	0.99–2.91	0.05
DFS	90	2.17	0.99–4.84	0.05	43	1.97	0.59–6.58	0.27	139	2.03	1.05–3.949	0.04
												
*Cox regression*												
OS	90	1.18	0.58–2.39	0.62	39	5.23	1.13–24.18	0.04	130	1.39	0.85–2.64	0.26
DFS	85	1.78	0.76–4.17	0.17	36	1.84	0.21–15.77	0.57	129	1.59	0.81–3.15	0.19

Abbreviations: 95% CI=95% confidence interval; DFS=disease-free survival; HR=hazard ratio; n=number of cases analysed; OS=overall survival.
